# Bloodstream Infection Risk, Incidence, and Deaths for Hospitalized Patients during Coronavirus Disease Pandemic

**DOI:** 10.3201/eid2710.210538

**Published:** 2021-10

**Authors:** Bhavarth S. Shukla, Prem R. Warde, Eric Knott, Sebastian Arenas, Darryl Pronty, Reinaldo Ramirez, Arely Rego, Miriam Levy, Martin Zak, Dipen J. Parekh, Tanira Ferreira, Hayley B. Gershengorn

**Affiliations:** University of Miami Health System, Miami, Florida, USA (B.S. Shukla, P.R. Warde, S. Arenas, D. Pronty, R. Ramirez, A. Rego, M. Levy, D.J. Parekh, T. Ferreira, H.B. Gershengorn);; University of Miami Miller School of Medicine, Miami (B.S. Shukla, E. Knott, M. Zak, D.J. Parekh, T. Ferreira, H.B. Gershengorn);; Albert Einstein College of Medicine, Bronx, New York, USA (H.B. Gershengorn)

**Keywords:** coronavirus disease, COVID-19, pandemic, severe acute respiratory coronavirus 2, SARS-CoV-2, coronaviruses, viruses, respiratory infections, bloodstream infection risk, prone positioning, incidence, deaths, hospitalized patients, infection control, zoonoses

## Abstract

Hospital-acquired infections are emerging major concurrent conditions during the coronavirus disease (COVID-19) pandemic. We conducted a retrospective review of hospitalizations during March‒October 2020 of adults tested by reverse transcription PCR for severe acute respiratory syndrome coronavirus 2. We evaluated associations of COVID-19 diagnosis with risk for laboratory-confirmed bloodstream infections (LCBIs, primary outcome), time to LCBI, and risk for death by using logistic and competing risks regression with adjustment for relevant covariates. A total of 10,848 patients were included in the analysis: 918 (8.5%) were given a diagnosis of COVID-19, and 232 (2.1%) had LCBIs during their hospitalization. Of these patients, 58 (25%) were classified as having central line‒associated bloodstream infections. After adjusting for covariates, COVID-19‒positive status was associated with higher risk for LCBI and death. Reinforcement of infection control practices should be implemented in COVID-19 wards, and review of superiority and inferiority ranking methods by National Healthcare Safety Network criteria might be needed.

The incidence of co-infection with either bacterial or fungal pathogens in patients hospitalized because of coronavirus disease (COVID-19) during the ongoing pandemic has become a topic of great interest. Hospitalized COVID-19 patients have shown co-infection rates as low as 7% (*1*) and as high as 15%, and as many as 27% of those who ultimately die are co-infected (*1**–**5*). Although some COVID-19 patients have bacterial or fungal co-infections, it appears that nosocomial origins for co-infection might be a major factor. One study found that only 3.2% of hospitalized COVID-19 patients were co-infected at the time of hospital admission (*3*), and another study demonstrated a cumulative risk of 25% of developing a bloodstream infection in critically ill COVID-19 patients, but only after 48 hours in the intensive care unit (ICU) (*6*).

Sparse evidence exists that directly compares nosocomial incidence of bloodstream infection in those having COVID-19 with other hospitalized populations. A multicenter study in New York, New York, USA, found bloodstream infections in only 3.8% of hospitalized patients who had COVID-19 but in 8.0% of patients who did not have COVID-19 (*7*). When comparing with patients who had influenza, Hughes et al. found a 1.8-fold increased rate of bloodstream infection in COVID-19 patients (2.5% vs. 1.4%) hospitalized in the United Kingdom (*3*). However, differences in the types of case-patients by COVID-19 status were not considered in either study. Moreover, the generalizability of these differences by COVID-19 status to other geographic regions remains unknown.

Little evidence exists for risk factors for nosocomial infection in COVID-19. A single-center study from Wuhan, China, identified an association related to use of invasive devices and combination antimicrobial drugs, as well as having diabetes mellitus, with an increased risk for developing a hospital-acquired infection (HAI) (*8*). However, the external validity of these associations has not been explored.

In this study, we sought to investigate whether being infected with COVID-19 was independently associated with an increase in odds of developing a laboratory-confirmed bloodstream infection (LCBI). We also aimed to identify other potential risk factors for LCBI in hospitalized COVID-19 patients. We hypothesized that COVID-19 patients would have greater odds of acquiring an LCBI than hospitalized patients without COVID-19 after adjusting for relevant confounders, and that other risk factors might also be identified, which might serve as targets for interventions to reduce co-infection rates in this vulnerable group.

## Methods

### Study Design and Cohort

We conducted a retrospective cohort study of adult hospitalizations during March 25‒October 27, 2020, at an academic, tertiary, acute-care facility in Miami, Florida, USA, which lacks capacity to give care with extracorporeal membrane oxygenation. Patients were included in the cohort if they had >1 reverse transcription PCR completed; patients could be included more than once if they were admitted to the hospital more than once over the study period. During the study period, all patients were screened by reverse transcription PCR before hospital admission. Although there were no specific exclusion criteria, the facility does not offer pediatric or obstetric services, so pregnant woman and patients <18 years of age were not included. A restricted cohort of patients that had central venous catheters at any point during their hospital stay was also considered.

### Exposure and Outcomes

Our exposure of interest was COVID-19 positivity (determined by SARS-CoV-2 PCR testing) during the hospital stay. Patients who had >1 positive test result (from 7 days before admission up until discharge with or without preceding negative test results) were considered positive for COVID-19. Our primary outcome was LCBI. Secondary outcomes were death, time to LCBI (time from hospital admission to first positive blood culture per patient admission), and development of central line‒associated bloodstream infection (CLABSI) evaluated by using the restricted cohort of patients who had a central venous catheter. We defined LCBIs and CLABSIs according to National Healthcare Safety Network (NHSN) 2020 criteria (*9*). In brief, LCBI is defined in these criteria as a single positive blood culture or molecular test result for a pathogen or 2 positive blood cultures for a commensal organism. CLABSI is defined as an LCBI associated with a central venous catheter in place for >2 calendar days (*9*).

### Data Sources and Variables

We obtained information for each patient from the hospital system’s electronic medical record by using EPIC software (https://www.epic.com). In addition to COVID-19 infection status and outcomes (including organism identification), we abstracted information on demographics (age, sex, race, primary insurance provider), organisms isolated from blood cultures, chronic health conditions, Elixhauser comorbidity conditions (*10*), body mass index, severity of acute illness (sequential organ failure assessment score [SOFA] during hospital day 1) (*11*), renal replacement therapy (either intermittent or continuous), invasive mechanical ventilation, care in the ICU, prone positioning (including persons using mechanical ventilation), central venous catheters, urinary catheters, systemic corticosteroids, tocilizumab, and remdesivir. For each of the resources other than prone positioning, we identified whether they were used and the total duration of use. For prone positioning, we were able to identify only use (not duration or timing of use). We also obtained data on hospital length of stay.

### Statistical Analysis and Ethics

We described the cohort by using standard summary statistics. We compared characteristics by outcome by using χ^2^ and Kruskal-Wallis tests as appropriate. Our primary analysis was a risk for LCBI assessment by using multivariable logistic regression modeling with an exposure of COVID-19 status. We included all data elements except prone positioning, remdesivir, and tocilizumab as covariables, and resource elements were modeled as receipt/nonreceipt before development of LCBI (or hospital discharge if no LCBI). To ensure our results would not be confounded by deaths in hospitals, we recreated the same models for hospital survivors and decedents separately.

To consider secondary outcomes, we first used multivariable Cox proportional hazards modeling with censoring at hospital discharge and a competing risk for death to assess the association of COVID-19 positivity and time to LCBI. We then constructed a multivariable logistic regression model to assess the association of COVID-19 positivity with risk for death by hospital discharge. For this model, we included days of resource use as covariates.

We also constructed 3 models to evaluate for LCBI that developed >2 calendar days from admission, indicated as LCBI HAI. Next, we reconstructed the models (for our primary and 2 secondary outcomes) by using the restricted cohort of patients who had used central venous catheters to assess risk for and time to CLABSI and death. Finally, to identify risk factors for infection among COVID-19 patients, we constructed 3 multivariable logistic regression models: for LCBI among all COVID-19 patients, for LCBI HAI among all COVID-19 patients, and for CLABSI among COVID-19 patients who had central venous catheters.

Because of a large number of patients who had missing data regarding calculation of SOFA, we imputed this score for each model (using multivariable regression modeling, including all covariates and outcome). We conducted 2 sensitivity analyses to assess the robustness of our primary analysis: only patients with an available SOFA score, and all patients but not including SOFA as a model covariate.

Study approval was obtained from the University of Miami Institutional Review Board (#20200739). We performed all analyses by using the programming language R (R Foundation for Statistical Computing, https://www.r-project.org). Results were considered significant if p<0.05. Because we did not adjust for multiple comparisons, we considered all nonprimary analyses to be hypothesis generating.

## Results

Our primary cohort consisted of 10,848 hospital admissions, of whom 918 (8.5%) were COVID-19 positive (Table 1; Appendix Figure 1). A total of 232 (2.1%) persons showed development of an LCBI: 64 (7.0%) of those who were COVID-19 positive and 168 (1.7%) of those who were COVID-19 negative. The subset of LCBIs acquired 2 calendar days after admission included 61 (95.3%) in the COVID-19‒positive patient group and 93 (58.4%) in the COVID-19 negative patient group (Appendix Figure 1). Evaluation of baseline characteristics showed major differences by bivariate analysis of the cohort when divided by outcome (Table 1) or COVID-19 status (Appendix Table 1), including sex, payer, comorbidity index, and SOFA score.

**Table 1:** Patients characteristics by outcome on bloodstream infection risk, incidence, and deaths for hospitalized patients during coronavirus disease pandemic, Miami, Florida, USA, March 25‒October 27, 2020*

Characteristic	Full cohort for LCBI analyses, no. (%)								Central line cohort for CLABSI analyses, no. (%)		
	NHSN LCBI				NHSN LCBI (HAI)						
	No LCBI	LCBI	p value†		No LCBI	LCBI	p value†		No CLABSI	CLABSI	p value†
Patient admissions	10,616 (97.9)	232 (2.1)			10,694 (98.6)	154 (1.4)			2,840 (98.0)	58 (2.0)	
COVID-19 RT-PCR positive	854 (8.0)	64 (28)	<0.001		857 (8.0)	61 (40)	<0.001		365 (13)	32 (55)	<0.001
Age, y	63 (52‒73)	66 (55‒74)	0.093		63 (52‒73)	66 (54‒74)	0.4		66 (55‒76)	66 (56‒72)	0.3
Sex‡			0.094				0.001				0.016
M	5,479 (52)	134 (58)	0.094		5,512 (52)	101 (66)	0.001		1,343 (47)	37 (64)	0.016
F	5,136 (48)	98 (42)			5,181 (48)	53 (34)			1,497 (53)	21 (36)	
Race/ethnicity			0.041				0.053				<0.001
Non-Hispanic White	2,318 (22)	33 (14)			2,330 (22)	21 (14)			553 (19)	7 (12)	
Non-Hispanic Black	280 (2.6)	3 (1.3)			281 (2.6)	2 (1.3)			76 (2.7)	1 (1.7)	
Hispanic White	5,152 (49)	123 (53)			5,188 (49)	87 (56)			1,338 (47)	33 (57)	
Hispanic Black	1,952 (18)	49 (21)			1,976 (18)	25 (16)			647 (23)	4 (6.9)	
Other	577 (5.4)	17 (7.3)			581 (5.4)	13 (8.4)			154 (5.4)	8 (14)	
Unknown	337 (3.2)	7 (3.0)			338 (3.2)	6 (3.9)			72 (2.5)	5 (8.6)	
Payer			<0.001				<0.001				0.077
Commercial	3,849 (36)	59 (25)			3,869 (36)	39 (25)			741 (26)	15 (26)	
Government	73 (0.7)	4 (1.7)			74 (0.7)	3 (1.9)			17 (0.6)	2 (3.4)	
Medicaid	1,248 (12)	35 (15)			1,254 (12)	29 (19)			407 (14)	12 (21)	
Medicare	4,964 (47)	130 (56)			5,014 (47)	80 (52)			1,597 (56)	27 (47)	
Other	482 (4.5)	4 (1.7)			483 (4.5)	3 (1.9)			78 (2.7)	2 (3.4)	
BMI, kg/m^2^§	27 (23‒31)	26 (23‒30)	0.2		27 (23‒31)	27 (23‒31)	0.7		26 (22‒31)	28 (25‒33)	0.028
Elixhauser comorbidity index	15 (4‒29)	27 (15‒40)	<0.001		15 (4‒29)	26 (15‒39)	<0.001		23 (11‒36)	22 (12‒34)	>0.9
Urethral catheter	3,406 (32)	126 (54)	<0.001		3,430 (32)	102 (66)	<0.001		1,249 (44)	45 (78)	<0.001
Central line	2,690 (25)	208 (90)	<0.001		2,754 (26)	144 (94)	<0.001		NA	NA	NA
Mechanical ventilation	750 (7.1)	97 (42)	<0.001		767 (7.2)	80 (52)	<0.001		569 (20)	39 (67)	<0.001
Steroid treatment	3,094 (29)	127 (55)	<0.001		3,119 (29)	102 (66)	<0.001		1,155 (41)	49 (84)	<0.001
ICU admission	2,043 (19)	135 (58)	<0.001		2,067 (19)	111 (72)	<0.001		1,118 (39)	48 (83)	<0.001
Dialysis	657 (6.2)	82 (35)	<0.001		682 (6.4)	57 (37)	<0.001		312 (11)	29 (50)	<0.001
SOFA score¶	1 (0‒3)	3 (2‒5)	<0.001		1 (0‒3)	3 (2‒5)	<0.001		2 (1‒4)	3 (2‒4)	0.055
Central line duration, d	0.0 (0.0‒0.3)	14.2 (5.0‒28.0)	<0.001		0.0 (0.0‒0.4)	20.8 (10.2‒34.3)	<0.001		5 (3‒11)	28 (15‒54)	<0.001
Hospital LOS, d	3.5 (1.9‒6.9)	18.8 (9.4‒30.9)	<0.001		3.5 (1.9‒6.9)	24.9 (14.3‒36.7)	<0.001		8 (5‒14)	29 (20‒50)	<0.001
Deaths in hospital	258 (2.4)	50 (22)	<0.001		267 (2.5)	41 (27)	<0.001		201 (7.1)	21 (36)	<0.001

*Values are no. (%) or median (IQR). BMI, body mass index; CLABSI, central line‒associated bloodstream infection; COVID-19, coronavirus disease; HAI, healthcare associated infection; IQR, interquartile range; LCBI, laboratory-confirmed bloodstream infection; LOS, length of stay; NA, not applicable; NHSN, National Healthcare Safety Network; RT-PCR, reverse transcription PCR; SOFA. sequential organ failure assessment. †Statistical tests performed: Fisher exact test; Wilcoxon rank-sum test; χ^2^ test of independence. ‡One patient of an unknown sex in the full cohort (did not have an LCBI). §Eight patients had missing BMIs in the full cohort (3 in the CLABSI cohort); none had a bloodstream infection. ¶Indicates patients who had missing SOFA scores: 4,815 (55%) of no LCBI full cohort, 104 (55%) of LCBI full cohort, 4,851 (55%) of no LCBI-HAI full cohort, 68 (56%) of LCBI-HAI full cohort, 1,355 (52%) of no CLABSI, and 25 (57%) of CLABSI patients.

Organisms most frequently cultured meeting NHSN definitions for LCBI among COVID-19 patients were *Candida* spp. (n = 11, 17.2%), *Enterococcus faecalis* (n = 8, 12.5%), and *Staphylococcus epidermidis* (7, 10.9%). These organisms were also found in the context of polymicrobial cultures (internally defined as >2 organisms isolated from the bloodstream within a 48-hour period). They constituted the largest percentage of the cohort of LCBI at 28.1% (n = 18) (Figure). Similar organisms were observed with cultures from COVID-19 patients meeting NHSN definition for CLABSI: *Candida* spp. 50.0% (n = 16), *E. faecalis* 25.0% (n = 8), and *S. epidermis* 12.5% (n = 4). The organisms identified on blood culture from COVID-19‒positive versus COVID-19‒negative patients for LCBI and CLABSI were comparatively different, but because of low numbers, no statistical analysis was performed (Appendix Figure 2).

**Figure:**
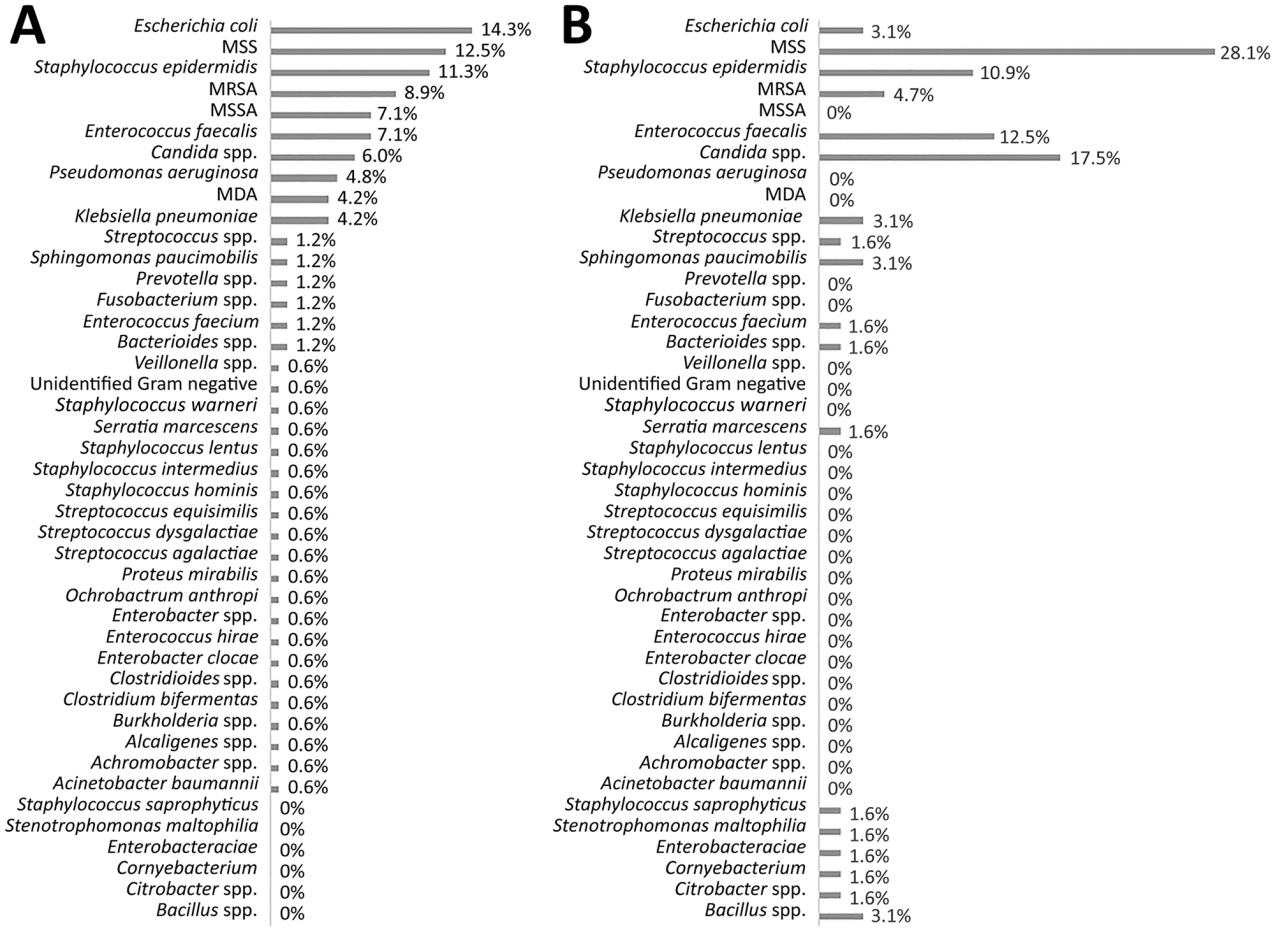
Organisms responsible for laboratory-confirmed bloodstream infections during COVID-19 pandemic, Miami, Florida, USA, March 25‒October 27, 2020. A) COVID-19‒negative patients (n = 168). B) COVID-19‒positive patients (n = 64). COVID-19, coronavirus disease; MDA, organisms isolated during admission (defined as >2 organisms isolated from the bloodstream >48 hours apart during admission); MRSA, methicillin-resistant *Staphylococcus aureus*; MSSA, methicillin-sensitive *S. aureus*; MSS, multiple organisms isolated during bloodstream infection episode (defined >2 organisms isolated from the bloodstream within a 48-hour period from the index isolate).

### Association of COVID-19 Status with Outcomes

After adjusting for potential confounders, we found that COVID-19 positivity was associated with an increase in odds of an LCBI developing (odds ratio [OR] 3.88, 95% CI 2.70–5.51; p<0.001 (Table 2; Appendix Table 2). COVID-19 was also significantly associated with LCBI development for either hospital survivors (OR 3.50, 95% CI 2.28–5.27; p<0.001) or decedents (OR 3.14, 95% CI 1.33–7.72; p = 0.01) (Appendix Table 3) when considered separately. Our results were robust to sensitivity analyses aimed at addressing missing SOFA scores (Appendix Tables 4, 5).

**Table 2:** Adjusted association for virus positivity with outcomes for patients during the coronavirus disease pandemic, Miami, Florida, USA, March 25‒October 27, 2020*

Cohort	Primary outcome	OR/HR (95% CI)
Full	Risk for LCBI†	3.88 (2.70‒5.51)
	Time to LCBI‡	2.35 (1.77‒3.13)
	Risk for death,§ adjusted for LCBI	6.68 (4.94‒9.01)
	Risk for LCBI-HAI†	5.58 (3.67‒8.43)
	Time to LCBI-HAI‡	2.73 (1.94‒3.85)
	Risk for death,§ adjusted for LCBI-HAI	6.64 (4.91‒8.96)
Central line	Risk for CLABSI†	5.68 (2.94‒11.1)
	Time to CLABSI‡	2.86 (1.75‒4.65)
	Risk for death§	5.30 (3.68‒7.64)

*All p values were <0.001. BMI, body mass index; CLABSI: central line‒associated bloodstream infection; HR, hazard ratio; ICU, intensive care unit; LCBI, laboratory-confirmed bloodstream infection; OR, odds ratio; SOFA, sequential organ failure assessment. †Modeled with logistic regression including age, sex, race/ethnicity, payer, BMI, no. comorbidities, previous urethral catheter use, previous central line use, previous mechanical ventilation, previous steroid treatment, previous ICU admission, previous dialysis, previous prone positioning, previous remdesivir treatment, tocilizumab treatment, and imputed SOFA score as covariates. OR is reported. ‡Modeled with proportional hazards model including age, sex, race/ethnicity, payer, BMI, no. comorbidities, previous urethral catheter use, previous central line use, previous mechanical ventilation, previous steroid treatment, previous ICU admission, previous dialysis, and imputed SOFA score as covariates. HR is reported. §Modeled with logistic regression including age, sex, race/ethnicity, payer, BMI, no. comorbidities, urethral catheter days, central line days, previous mechanical ventilation days, steroid treatment days, ICU days, dialysis days , imputed SOFA score, and LCBI as covariates; OR is reported.

We found significant associations with regards to time to LCBI (hazard ratio 2.35, 95% CI 1.77–3.13; p<0.001) (Table 2; Appendix Table 2, Figure 3). COVID-19 positivity was associated with an increased odds of hospital death (OR 6.68, 95% CI 4.94–9.01; p<0.001). After restricting the cohort to patients with positive cultures after 2 calendar days, we found that COVID-19 was associated with LCBI-HAI; after restricting the cohort to patients with central lines, we found that COVID-19 was associated with CLABSI (Table 2; Appendix Tables 6, 7).

### Non‒COVID-19 Risk Factors for LCBI, LCBI HAI, and CLABSI

In a subgroup analysis of only COVID-19 patients, we found that previous central line use was associated with an increased risk for LCBI (OR 8.11, 95% CI 2.40–37.3; p = 0.002) and LCBI HAI (OR 11.7, 95% CI 2.94–78.2; p = 0.002) (Table 3). We found no major associations with use of remdesivir, steroids, or tocilizumab. Another finding in the subgroup analysis was that prone positioning did not have any major associations with risk for outcomes in patients who had COVID-19.

**Table 3:** Subgroup analysis of clinical variables in patients who had COVID-19 and bloodstream infection risk, incidence, and deaths for hospitalized patients during coronavirus disease pandemic, Miami, Florida, USA, March 25‒October 27, 2020*

Characteristic	LCBI			LCBI HAI			CLABSI	
	OR (95% CI)	p value		OR (95% CI)	p value		OR (95% CI)	p value
Sex								
M	1.84 (0.96‒3.61)	0.068		2.09 (1.07‒4.23)	0.034		3.50 (1.29‒10.7)	0.019
F	0.54 (0.28‒1.04)	0.068		0.48 (0.24‒0.93)	0.034		0.29 (0.09‒0.77)	0.019
Age, y	0.97 (0.94‒1.00)	0.03		0.97 (0.94‒1.00)	0.029		0.99 (0.94‒1.03)	0.6
BMI, kg/m^2^	0.99 (0.94‒1.04)	0.7		0.99 (0.95‒1.05)	0.8		0.98 (0.91‒1.05)	0.5
Comorbidity index	1.00 (0.98‒1.03)	0.9		1.00 (0.97‒1.02)	0.8		0.97 (0.93‒1.00)	0.094
Previous urethral catheter	1.96 (0.75‒5.18)	0.2		1.99 (0.74‒5.41)	0.2		1.60 (0.28‒10.2)	0.6
Previous central line	8.11 (2.40‒37.3)	0.002		11.7 (2.94‒78.2)	0.002		NA	NA
Previous mechanical ventilation	2.82 (0.91‒9.89)	0.086		2.18 (0.70‒7.44)	0.2		∞ (0.00‒∞)	>0.9
Previous steroid treatment	0.91 (0.40‒2.14)	0.8		0.91 (0.39‒2.21)	0.8		1.42 (0.35‒6.73)	0.6
Previous ICU admission	2.47 (0.74‒7.61)	0.12		3.56 (1.07‒11.5)	0.034		0.00 (0.00‒∞)	>0.9
Previous dialysis	1.01 (0.46‒2.14)	>0.9		0.95 (0.43‒2.07)	>0.9		0.59 (0.20‒1.68)	0.3
Prone positioning	1.09 (0.49‒2.37)	0.8		1.21 (0.55‒2.69)	0.6		2.02 (0.64‒6.97)	0.2
Remdesivir treatment	1.58 (0.78‒3.24)	0.2		1.58 (0.76‒3.32)	0.2		1.29 (0.45‒3.77)	0.6
Tocilizumab treatment	1.29 (0.42‒3.77)	0.6		1.23 (0.39‒3.62)	0.7		1.10 (0.25‒4.44)	0.9
SOFA score imputed	1.00 (0.86‒1.14)	>0.9		0.96 (0.82‒1.10)	0.6		0.083 (0.63‒1.03)	0.12

*Model also adjusted for race/ethnicity and payer (these variables had no major association with outcomes). BMI, body mass index; CLABSI, central line‒associated bloodstream infection; ICU, intensive care unit; LCBI, laboratory‒confirmed bloodstream infection; NA, not applicable; SOFA, sequential organ failure assessment.

## Discussion

Before the COVID-19 pandemic, HAIs were well-recognized as a cause of death (*12*). To date, only a few studies have evaluated the effect of the COVID-19 pandemic on HAIs and their outcomes, particularly LCBIs (*3*,*13*). Using data for >10,000 patients hospitalized after SARS-CoV-2 testing, we found that COVID-19 positivity was associated with a 3.88-fold increased odds of developing an LCBI. This finding might be related to COVID-19 itself or other variables not accounted for in our cohort, such as changes in supplementary nursing care or changes in infection control practices associated with the care of these patients. In addition, isolates responsible for LCBI and CLABSI in COVID-19 patients versus non‒COVID-19 patients show major differences with regards to type and number of organisms.

Prone positioning has proven benefits for patients who have non-COVID-19‒associated acute respiratory distress syndrome requiring invasive mechanical ventilation (*14*). Studies have noted increases in use of prone positioning as treatment for critical care patients who have influenza (*15*) and, in recent months, data have emerged suggesting benefits of prone positioning for ventilated patients (*16**–**20*) and nonventilated patients who have COVID-19 (*21*). Although potential adverse effects, such as pressure ulcers (*22*) and deep venous thromboses (*23*), have been observed with prone positioning, we did not find any statistical association with our primary outcomes.

The strengths of our study stem from detailed clinical data (including organism identification) and severity of illness information (both acute and chronic) available to us. Our study is limited by a high rate of missing SOFA score data. However, the robustness of our results to sensitivity analyses, in which we excluded patients who had missing SOFA data and excluded SOFA as a model covariate, suggests that this limitation had minimal effect on our findings. Although our sample included >10,000 patients (of whom 918 patients had COVID-19), we included only patients from a single hospital, which might limit the generalizability of our results. In addition, several of the factors included in our models occurred after COVID-19 testing (our exposure), making it plausible that these factors are mediators rather than confounders of the association of COVID-19 with outcomes.

Another limitation of the study was our inability to include admission symptoms or central venous catheter insertion sites in the analysis. This limitation was largely caused by inconsistent documentation of these data points in a nondiscrete format in our electronic medical record. We also were not able to address markers of hospital operational stressors that might have varied over the time period of our study and might effect patient outcomes. Collinearity of clinical variables included in the models was also a concern. However, our evaluation identified only 2 variables that had higher correlation coefficients (previous mechanical ventilation and ICU stay) (Appendix Figure 4). A final limitation was the difficulty in analyzing dose and type of steroids and antimicrobial drugs given before and after bloodstream infections, as well as timing and duration of prone positioning.

As more data emerge regarding increases in HAIs during the COVID-19 pandemic (*24*), we propose that these challenges warrant reevaluation of the NHSN SIR methods for LCBI and CLABSI in COVID-19‒designated care areas. Further studies are needed to clarify the relationship between COVID-19 and non-LCBI infections to ascertain whether prone positioning, COVID-19-specific treatments, changes in adherence to infection control practices, or a combination of these variables might be associated with higher rates of other HAIs.

In conclusion, inpatient management of patients who have COVID-19 has brought many changes in treatment protocols and associated challenges, including adherence to infection control best practices. Established infection control best practices should be reemphasized among COVID-19 patients who might be at higher risk for LCBI, adding a concurrent condition to an already vulnerable population.

Appendix Additional information on bloodstream infection risk, incidence, and deaths for hospitalized patients during coronavirus disease pandemic.
