# Supplementary material for: Bloodstream Infection Risk, Incidence, and Deaths for Hospitalized Patients during Coronavirus Disease Pandemic
Source: Emerg Infect Dis. 2021 Oct;27(10):2588–94. doi: 10.3201/eid2710.210538 (PMC8462325; doi:10.3201/eid2710.210538)
Supplement: Appendix — Additional information on bloodstream infection risk, incidence, and deaths for hospitalized patients during coronavirus disease pandemic. [file 21-0538-Techapp-s1.pdf]

Article DOI: https://doi.org/10.3201/eid2710.210538

Bloodstream Infection Risk, Incidence, and Deaths for Hospitalized Patients During Coronavirus Disease Pandemic

Appendix

Appendix Table 1. Cohort characteristics by COVID-19 positivity*

Characteristic	COVID-19 Negative, n = 9,930	COVID-19 Positive , n = 918	p value†
Age	63 (52, 73)	69 (56, 80)	<0.001
Sex‡			0.11
F	4,792 (48%)	442 (48%)	
M	5,138 (52%)	475 (52%)	
Race/ethnicity			<0.001
Non-Hispanic White	2,256 (23%)	95 (10%)	
Non-Hispanic Black	253 (2.5%)	30 (3.3%)	
Hispanic White	4,753 (48%)	522 (57%)	
Hispanic Black	1,835 (18%)	166 (18%)	
Other	520 (5.2%)	74 (8.1%)	
Unknown	313 (3.2%)	31 (3.4%)	
Payer			<0.001
Commercial	3,691 (37%)	217 (24%)	
Government	33 (0.3%)	44 (4.8%)	
Medicaid	1,193 (12%)	90 (9.8%)	
Medicare	4,588 (46%)	506 (55%)	
Other	425 (4.3%)	61 (6.6%)	
BMI§	27 (23, 31)	28 (24, 32)	<0.001
Elixhauser comorbidity index	15 (4, 30)	13 (4, 24)	<0.001
Urethral catheter	3,294 (33%)	238 (26%)	<0.001
Central line	2,501 (25%)	397 (43%)	<0.001
Mechanical ventilation	687 (6.9%)	160 (17%)	<0.001
Steroid treatment	2,685 (27%)	536 (58%)	<0.001
ICU admission	1,951 (20%)	227 (25%)	<0.001
Dialysis	637 (6.4%)	102 (11%)	<0.001
SOFA score¶	1 (0, 3)	2 (1, 4)	<0.001
Central line duration, d	0.0 (0.0, 0.2)	0.0 (0.0, 7.3)	<0.001
Hospital LOS, d	3.4 (1.9, 6.5)	7.0 (3.2, 14.2)	<0.001
Hospital deaths	167 (1.7%)	141 (15%)	<0.001

*Values are no. (%) or median (IQR). BMI, body mass index; COVID-19, coronavirus disease; CLABSI, central line–associated bloodstream infection; IQR, interquartile range; LCBI, laboratory-confirmed bloodstream infection; LOS, length of stay; SOFA: sequential organ failure assessment.
†Statistical tests performed: Wilcoxon rank-sum test, Fisher exact test for count data with simulated p value (based on 2,000 replicates); and Fisher exact test for count data.
‡One patient whose sex was unknown in the full cohort (did not have an LCBI).
§Eight patients who had a missing BMI in the full cohort (3 in the CLABSI cohort); none had a bloodstream infection.
¶Indicates patients who had missing SOFA scores: 4,815 (55%) of no LCBI full cohort, 104 (55%) of LCBI full cohort, 4,851 (55%) of no LCBI-HAI full cohort, 68 (56%) of LCBI-HAI full cohort, 1,355 (52%) of no CLABSI, and 25 (57%) of CLABSI patients.

Appendix Table 2. Full model for assessing association of COVID-19 positivity with outcomes with SOFA imputed (primary analysis), LCBI*

	Risk for infection			Time to infection			Risk for death		
Characteristic	OR	95% CI	p value†	HR	95% CI	p value†	OR	95% CI	p value†
COVID-19 RT-PCR positive	3.88	2.70, 5.51	<0.001	2.35	1.77, 3.13	<0.001	6.68	4.94, 9.01	<0.001
Male sex	1.27	0.96, 1.67	0.093	1.24	0.96, 1.61	0.11	1.07	0.82, 1.40	0.6
Race/ethnicity									
Non-Hispanic White	–	–		–	–		–	–	
Hispanic Black	0.65	0.15, 1.88	0.5	0.61	0.19, 2.03	0.4	2.04	0.91, 4.22	0.067
Hispanic White	1.45	0.98, 2.19	0.073	1.49	1.01, 2.20	0.044	1.47	1.00, 2.20	0.053
Non-Hispanic Black	1.37	0.86, 2.22	0.2	1.33	0.83, 2.13	0.2	1.82	1.15, 2.91	0.012
Other	1.66	0.87, 3.05	0.11	1.74	0.99, 3.06	0.056	1.46	0.77, 2.67	0.2
Unknown	1.53	0.60, 3.41	0.3	1.76	0.79, 3.94	0.2	0.93	0.38, 2.10	0.9
Payer									
Commercial	–	–		–	–		–	–	
Government	1.17	0.32, 3.37	0.8	0.89	0.33, 2.44	0.8	1.44	0.44, 4.01	0.5
Medicaid	1.48	0.94, 2.30	0.085	1.23	0.81, 1.87	0.3	1.15	0.66, 1.94	0.6
Medicare	1.31	0.90, 1.93	0.2	1.31	0.89, 1.92	0.2	0.79	0.53, 1.19	0.3
Other	0.56	0.17, 1.43	0.3	0.63	0.23, 1.74	0.4	4.85	2.85, 8.17	<0.001
BMI	1.00	0.97, 1.02	0.7	1.00	0.98, 1.02	0.8	1.04	1.02, 1.06	<0.001
Age	0.99	0.98, 1.00	0.029	0.99	0.98, 1.00	0.017	1.04	1.03, 1.06	<0.001
Elixhauser comorbidity index	1.03	1.02, 1.04	<0.001	1.02	1.01, 1.03	<0.001	1.03	1.02, 1.04	<0.001
Previous urethral catheter	1.02	0.72, 1.44	>0.9	0.86	0.63, 1.18	0.4			
Previous central line	2.61	1.90, 3.61	<0.001	1.29	0.97, 1.73	0.085			
Previous mechanical ventilation	1.44	0.92, 2.26	0.11	1.11	0.72, 1.73	0.6			
Previous steroid treatment	0.78	0.57, 1.05	0.1	0.57	0.43, 0.75	<0.001			
Previous ICU admission	1.79	1.19, 2.66	0.004	1.46	1.00, 2.12	0.05			
Previous dialysis	2.33	1.62, 3.31	<0.001	1.50	1.04, 2.15	0.029			
SOFA score, imputed	1.05	0.99, 1.11	0.073	1.08	1.02, 1.14	0.01	1.31	1.24, 1.37	<0.001
Urethral catheter days							1.00	0.97, 1.02	0.8
Central line days							0.98	0.97, 1.00	0.033
Ventilator days							1.01	0.98, 1.03	0.5
Steroid treatment days							1.03	1.00, 1.05	0.022
ICU admission days							1.09	1.06, 1.12	<0.001
Dialysis days							1.00	0.98, 1.02	>0.9
NHSN LCBI							3.19	1.96, 5.09	<0.001

*BMI, body mass index; CLABSI: central line–associated bloodstream infection; COVID-19, coronavirus disease; HR, hazard ratio; ICU, intensive care unit; IQR, interquartile range; LCBI, laboratory-confirmed bloodstream infection; LOS, length of stay; NHSN, National Healthcare Safety Network; RT-PCR, reverse transcription PCR; SOFA, sequential organ failure assessment; –, not available.
†Statistical tests performed: Fisher exact test; Wilcoxon rank-sum test, and χ^2 test of independence.

Appendix Table 3. Full model for assessing association of COVID-19 positivity with development of LCBI stratified by survival status*

	Died			Survived		
Characteristic	OR	95% CI	p value†	OR	95% CI	p value†
COVID-19 RT-PCR positive	3.14	1.33, 7.72	0.01	3.50	2.28, 5.27	<0.001
Male sex	1.14	0.56, 2.35	0.7	1.28	0.95, 1.75	0.11
Race/ethnicity						
Non-Hispanic White	–	–		–	–	
Hispanic Black	0.00	0.00, ∞	>0.9	0.79	0.19, 2.31	0.7
Hispanic White	1.79	0.62, 6.09	0.3	1.40	0.91, 2.20	0.14
Non-Hispanic Black	0.83	0.18, 3.72	0.8	1.43	0.86, 2.40	0.2
Other	3.24	0.75, 14.7	0.12	1.49	0.69, 3.01	0.3
Unknown	7.77	1.26, 51.2	0.027	0.83	0.19, 2.41	0.8
Payer						
Commercial	–	–		–	–	
Government	0.81	0.08, 5.64	0.8	1.05	0.16, 4.07	>0.9
Medicaid	1.04	0.27, 3.67	>0.9	1.49	0.90, 2.42	0.11
Medicare	1.07	0.41, 2.89	0.9	1.38	0.91, 2.13	0.14
Other	0.10	0.00, 0.83	0.066	0.63	0.15, 1.77	0.4
BMI	1.03	0.99, 1.08	0.2	0.98	0.96, 1.01	0.2
Age	0.97	0.93, 1.00	0.049	0.99	0.98, 1.00	0.026
Elixhauser comorbidity index	1.04	1.01, 1.07	0.018	1.03	1.02, 1.04	<0.001
Previous urethral catheter	1.27	0.46, 3.70	0.7	0.88	0.59, 1.28	0.5
Previous central line	3.12	1.02, 11.3	0.061	2.43	1.73, 3.42	<0.001
Previous mechanical ventilation	0.55	0.20, 1.66	0.3	1.54	0.92, 2.60	0.1
Previous steroid treatment	0.61	0.27, 1.40	0.2	0.75	0.53, 1.05	0.1
Previous ICU admission	0.99	0.29, 3.25	>0.9	1.81	1.16, 2.76	0.008
Previous dialysis	0.81	0.37, 1.74	0.6	2.68	1.77, 4.00	<0.001
SOFA score, imputed	0.99	0.88, 1.11	>0.9	1.06	0.99, 1.14	0.078

*BMI, body mass index; CLABSI, central line–associated bloodstream infection; COVID-19, coronavirus disease; HR, hazard ratio; ICU, intensive care unit; IQR, interquartile range; LCBI, laboratory-confirmed bloodstream infection; LOS, length of stay; RT-PCR, reverse transcription PCR; SOFA, sequential organ failure assessment; –, not available.

†Statistical tests performed: Fisher exact test, Wilcoxon rank-sum test, and χ^2 test of independence.

Appendix Table 4. Full model for assessing association of COVID-19 positivity with outcomes in sensitivity analysis using only cases that had available SOFA scores, LCBI*

	Risk for infection			Time to infection			Risk for death		
Characteristic	OR	95% CI	p value†	HR	95% CI	p value†	OR	95% CI	p value†
COVID-19 RT-PCR positive	3.90	2.40, 6.25	<0.001	2.62	1.79, 3.83	<0.001	6.77	4.64, 9.87	<0.001
Male sex	1.45	1.00, 2.13	0.052	1.50	1.05, 2.14	0.027	1.01	0.72, 1.41	>0.9
Race/ethnicity									
Non-Hispanic White	–	–		–	–		–	–	
Hispanic Black	0.35	0.02, 1.78	0.3	0.31	0.04, 2.52	0.3	2.89	1.06, 7.06	0.026
Hispanic White	1.46	0.88, 2.52	0.2	1.53	0.92, 2.56	0.1	1.52	0.93, 2.56	0.1
Non-Hispanic Black	1.02	0.53, 1.98	>0.9	1.05	0.54, 2.03	0.9	2.20	1.24, 4.00	0.008
Other	1.75	0.75, 3.85	0.2	1.62	0.76, 3.45	0.2	1.37	0.60, 2.97	0.4
Unknown	1.04	0.24, 3.21	>0.9	1.18	0.38, 3.71	0.8	0.39	0.09, 1.29	0.2
Payer									
Commercial	–	–		–	–		–	–	
Government	2.74	0.71, 8.49	0.1	2.18	0.87, 5.42	0.1	1.16	0.23, 4.29	0.8
Medicaid	1.67	0.92, 2.97	0.086	1.32	0.77, 2.27	0.3	1.44	0.73, 2.72	0.3
Medicare	1.05	0.63, 1.78	0.8	1.07	0.63, 1.81	0.8	0.80	0.48, 1.33	0.4
Other	0.56	0.13, 1.71	0.4	0.57	0.17, 1.92	0.4	3.43	1.66, 6.91	<0.001
BMI	1.00	0.98, 1.03	0.8	1.01	0.98, 1.03	0.5	1.03	1.00, 1.05	0.051
Age	0.99	0.98, 1.01	0.2	0.99	0.97, 1.00	0.094	1.04	1.02, 1.06	<0.001
Elixhauser comorbidity index	1.04	1.03, 1.05	<0.001	1.03	1.02, 1.04	<0.001	1.03	1.02, 1.04	<0.001
Previous urethral catheter	0.98	0.61, 1.56	>0.9	0.87	0.56, 1.36	0.5			
Previous central line	2.30	1.49, 3.55	<0.001	1.15	0.77, 1.72	0.5			
Previous mechanical ventilation	1.19	0.65, 2.18	0.6	0.88	0.49, 1.58	0.7			
Previous steroid treatment	0.74	0.48, 1.13	0.2	0.51	0.35, 0.76	<0.001			
Previous ICU admission	1.74	1.00, 2.94	0.042	1.37	0.81, 2.31	0.2			
Previous dialysis	1.42	0.83, 2.38	0.2	0.87	0.51, 1.47	0.6			
Urethral catheter days							0.99	0.95, 1.02	0.6
Central line days							0.96	0.94, 0.98	0.001
Ventilator days							1.02	0.98, 1.05	0.3
Steroid treatment days							1.03	1.00, 1.06	0.031
ICU admission days							1.12	1.09, 1.17	<0.001
Dialysis days							1.00	0.96, 1.03	0.8
NHSN LCBI							3.07	1.63, 5.67	<0.001
SOFA score	1.10	1.03, 1.18	0.007	1.13	1.06, 1.21	<0.001	1.33	1.25, 1.40	<0.001

*BMI, body mass index; CLABSI, central line–associated bloodstream infection; COVID-19, coronavirus disease; HR, hazard ratio; IQR, interquartile range; LCBI, laboratory-confirmed bloodstream infection; LOS, length of stay; NHSN, National Healthcare Safety Network; RT-PCR, reverse transcription PCR; SOFA, sequential organ failure assessment; –, not available.

†Statistical tests performed: Fisher exact test; Wilcoxon rank-sum test; and χ^2 test of independence.

Appendix Table 5. Full model for assessing association of COVID-19 positivity with outcomes in sensitivity analysis using all cases but without SOFA scores as a covariate, LCBI*

	Risk for infection			Time to infection			Risk for death		
Characteristic	OR	95% CI	p value†	HR	95% CI	p value†	OR	95% CI	p value†
COVID-19 RT-PCR positive	3.87	2.70, 5.49	<0.001	2.30	1.73, 3.05	<0.001	6.85	5.10, 9.18	<0.001
Male sex	1.27	0.97, 1.68	0.086	1.23	0.95, 1.60	0.11	1.15	0.89, 1.49	0.3
Race/ethnicity									
Non-Hispanic White	–	–		–	–		–	–	
Hispanic Black	0.65	0.15, 1.86	0.5	0.61	0.19, 2.00	0.4	1.92	0.85, 3.95	0.093
Hispanic White	1.45	0.98, 2.19	0.073	1.46	1.00, 2.15	0.052	1.50	1.03, 2.21	0.038
Non-Hispanic Black	1.36	0.85, 2.20	0.2	1.28	0.81, 2.04	0.3	1.77	1.13, 2.81	0.013
Other	1.64	0.86, 3.01	0.12	1.69	0.96, 2.96	0.069	1.64	0.89, 2.93	0.11
Unknown	1.51	0.59, 3.37	0.3	1.68	0.76, 3.75	0.2	0.95	0.39, 2.11	0.9
Payer									
Commercial	–	–		–	–		–	–	
Government	1.15	0.31, 3.33	0.8	0.91	0.33, 2.54	0.9	1.34	0.41, 3.68	0.6
Medicaid	1.48	0.94, 2.29	0.087	1.25	0.82, 1.90	0.3	1.12	0.64, 1.89	0.7
Medicare	1.32	0.90, 1.93	0.2	1.31	0.90, 1.92	0.2	0.81	0.55, 1.21	0.3
Other	0.61	0.18, 1.52	0.3	0.69	0.25, 1.89	0.5	6.12	3.72, 10.0	<0.001
BMI	1.00	0.97, 1.02	0.6	1.00	0.98, 1.02	0.7	1.04	1.02, 1.06	<0.001
Age	0.99	0.98, 1.00	0.036	0.99	0.98, 1.00	0.028	1.05	1.04, 1.06	<0.001
Elixhauser comorbidity index	1.03	1.02, 1.04	<0.001	1.02	1.01, 1.03	<0.001	1.04	1.03, 1.04	<0.001
Previous urethral catheter	1.02	0.72, 1.44	>0.9	0.86	0.63, 1.17	0.3			
Previous central line	2.64	1.92, 3.64	<0.001	1.32	0.98, 1.76	0.065			
Previous mechanical ventilation	1.52	0.98, 2.37	0.063	1.19	0.77, 1.83	0.4			
Previous steroid treatment	0.77	0.57, 1.04	0.1	0.57	0.43, 0.75	<0.001			
Previous ICU admission	1.81	1.20, 2.68	0.004	1.48	1.01, 2.15	0.042			
Previous dialysis	2.48	1.74, 3.50	<0.001	1.66	1.17, 2.34	0.004			
Urethral catheter days							1.00	0.98, 1.03	0.7
Central line days							0.98	0.96, 0.99	0.007
Ventilator days							1.01	0.99, 1.03	0.5
Steroid treatment days							1.03	1.01, 1.05	0.004
ICU admission days							1.09	1.06, 1.12	<0.001
Dialysis days							1.01	0.99, 1.03	0.4
NHSN LCBI							3.37	2.08, 5.35	<0.001

*BMI, body mass index; CLABSI, central line–associated bloodstream infection; COVID-19, coronavirus disease; HR, hazard ratio; IQR, interquartile range; LCBI, laboratory-confirmed bloodstream infection; LOS, length of stay; NHSN, National Healthcare Safety Network; RT-PCR, reverse transcription PCR; SOFA, sequential organ failure assessment; –, not available.

†Statistical tests performed: Fisher exact test; Wilcoxon rank-sum test; and χ^2 test of independence.

Appendix Table 6. Full model for assessing association of COVID-19 positivity with outcomes with SOFA imputed (primary analysis), LCBI-HAI*

	Risk for infection			Time to infection			Risk for death		
Characteristic	OR	95% CI	p value†	HR	95% CI	p value†	OR	95% CI	p value†
COVID-19 RT-PCR positive	5.58	3.67, 8.43	<0.001	2.73	1.94, 3.85	<0.001	6.64	4.91, 8.96	<0.001
Male sex	1.80	1.26, 2.59	0.001	1.71	1.22, 2.38	0.002	1.07	0.82, 1.39	0.6
Race/ethnicity									
Non-Hispanic White	–	–		–	–		–	–	
Hispanic Black	0.69	0.11, 2.45	0.6	0.61	0.14, 2.60	0.5	2.02	0.90, 4.18	0.071
Hispanic White	1.54	0.94, 2.61	0.095	1.55	0.96, 2.49	0.073	1.49	1.02, 2.24	0.045
Non-Hispanic Black	1.06	0.56, 1.99	0.9	1.01	0.56, 1.84	>0.9	1.85	1.16, 2.95	0.01
Other	1.70	0.78, 3.58	0.2	1.72	0.90, 3.27	0.1	1.50	0.79, 2.74	0.2
Unknown	2.00	0.69, 5.05	0.2	2.30	0.94, 5.62	0.069	0.93	0.38, 2.09	0.9
Payer									
Commercial	–	–		–	–		–	–	
Government	0.79	0.16, 2.79	0.7	0.63	0.20, 2.01	0.4	1.51	0.46, 4.18	0.5
Medicaid	1.90	1.11, 3.22	0.017	1.37	0.83, 2.25	0.2	1.13	0.65, 1.91	0.6
Medicare	1.25	0.77, 2.03	0.4	1.23	0.77, 1.97	0.4	0.79	0.53, 1.19	0.3
Other	0.74	0.17, 2.17	0.6	0.84	0.26, 2.71	0.8	4.75	2.79, 8.00	<0.001
BMI	1.00	0.97, 1.02	>0.9	1.00	0.98, 1.02	>0.9	1.04	1.02, 1.06	<0.001
Age	0.98	0.97, 0.99	0.005	0.98	0.97, 0.99	0.003	1.04	1.03, 1.06	<0.001
Elixhauser comorbidity index	1.03	1.02, 1.04	<0.001	1.01	1.00, 1.02	0.06	1.03	1.02, 1.04	<0.001
Previous urethral catheter	1.17	0.75, 1.82	0.5	0.94	0.63, 1.39	0.7			
Previous central line	4.98	3.17, 8.02	<0.001	1.71	1.12, 2.62	0.013			
Previous mechanical ventilation	1.39	0.84, 2.33	0.2	1.11	0.67, 1.86	0.7			
Previous steroid treatment	1.04	0.71, 1.51	0.9	0.68	0.48, 0.97	0.036			
Previous ICU admission	2.39	1.44, 3.90	<0.001	1.78	1.14, 2.80	0.012			
Previous dialysis	2.35	1.52, 3.60	<0.001	1.38	0.91, 2.08	0.13			
SOFA score, imputed	1.02	0.95, 1.09	0.7	1.05	0.98, 1.12	0.14	1.31	1.25, 1.37	<0.001
Urethral catheter days							1.00	0.97, 1.02	0.9
Central line days							0.98	0.97, 1.00	0.04
Ventilator days							1.01	0.98, 1.03	0.5
Steroid treatment days							1.03	1.00, 1.05	0.026
ICU admission days							1.09	1.06, 1.12	<0.001
Dialysis days							1.00	0.98, 1.02	>0.9
NHSN LCBI							2.80	1.58, 4.85	<0.001

*BMI, body mass index; CLABSI, central line–associated bloodstream infection; COVID-19, coronavirus disease; HR, hazard ratio; IQR, interquartile range; LCBI, laboratory-confirmed bloodstream infection; LOS, length of stay; NHSN, National Healthcare Safety Network; RT-PCR, reverse transcription PCR; SOFA, sequential organ failure assessment; –, not available.

†Statistical tests performed: Fisher exact test; Wilcoxon rank-sum test; and χ^2 test of independence.

Appendix Table 7. Full model for assessing association of COVID-19 positivity with outcomes with SOFA imputed (primary analysis), CLABSI*

	Risk for infection			Time to infection			Risk for death		
Characteristic	OR	95% CI	p value†	HR	95% CI	p value†	OR	95% CI	p value†
COVID-19 RT-PCR positive	5.68	2.94, 11.1	<0.001	2.86	1.75, 4.65	<0.001	5.30	3.68, 7.64	<0.001
Male sex	1.51	0.85, 2.75	0.2	1.45	0.85, 2.47	0.2	1.36	0.99, 1.88	0.061
Race/ethnicity									
Non-Hispanic White	–	–		–	–		–	–	
Hispanic Black	.12	0.06, 6.77	>0.9	0.91	0.12, 7.06	>0.9	2.24	0.82, 5.46	0.091
Hispanic White	1.48	0.66, 3.79	0.4	1.42	0.66, 3.06	0.4	1.55	0.98, 2.52	0.07
Non-Hispanic Black	0.45	0.11, 1.57	0.2	0.43	0.13, 1.44	0.2	1.73	0.99, 3.07	0.057
Other	2.32	0.75, 7.25	0.14	2.28	0.90, 5.79	0.084	1.82	0.88, 3.65	0.1
Unknown	5.41	1.43, 19.1	0.009	5.35	1.58, 18.0	0.007	1.51	0.55, 3.80	0.4
Payer									
Commercial	–	–		–	–		–	–	
Government	0.71	0.09, 3.62	0.7	0.54	0.08, 3.73	0.5	1.38	0.33, 5.19	0.6
Medicaid	1.89	0.80, 4.38	0.14	1.43	0.66, 3.09	0.4	0.67	0.35, 1.25	0.2
Medicare	1.10	0.50, 2.46	0.8	1.07	0.48, 2.38	0.9	0.61	0.38, 0.97	0.036
Other	1.39	0.20, 5.77	0.7	1.34	0.23, 7.75	0.7	1.01	0.37, 2.50	>0.9
BMI	0.99	0.96, 1.03	0.8	1.00	0.96, 1.04	>0.9	1.03	1.01, 1.05	0.01
Age	0.97	0.95, 0.99	0.012	0.97	0.95, 1.00	0.016	1.04	1.02, 1.05	<0.001
Elixhauser comorbidity index	1.01	0.99, 1.03	0.4	0.99	0.98, 1.01	0.4	1.02	1.01, 1.03	<0.001
Previous urethral catheter	1.31	0.59, 2.95	0.5	0.98	0.54, 1.79	>0.9			
Previous mechanical ventilation	2.51	1.04, 6.82	0.052	1.67	0.69, 4.04	0.3			
Previous steroid treatment	2.09	1.04, 4.42	0.045	1.35	0.65, 2.83	0.4			
Previous ICU admission	1.39	0.49, 3.66	0.5	1.27	0.49, 3.30	0.6			
Previous dialysis	2.07	1.05, 4.04	0.034	1.41	0.70, 2.85	0.3			
SOFA score, imputed	0.98	0.86, 1.09	0.7	0.99	0.89, 1.12	>0.9	1.21	1.14, 1.29	<0.001
Urethral catheter days							1.01	0.98, 1.03	0.6
Central line days							0.96	0.94, 0.98	<0.001
Ventilator days							1.03	1.00, 1.05	0.022
Steroid treatment days							1.02	1.00, 1.04	0.048
ICU admission days							1.07	1.04, 1.10	<0.001
Dialysis days							1.01	0.99, 1.03	0.4
NHSN LCBI							1.57	0.69, 3.46	0.3

*BMI, body mass index; CLABSI, central line–associated bloodstream infection; COVID-19, coronavirus disease; HR, hazard ratio; IQR, interquartile range; LCBI, laboratory-confirmed bloodstream infection; LOS, length of stay; NHSN, National Healthcare Safety Network; RT-PCR, reverse transcription PCR; SOFA, sequential organ failure assessment; –, not available.
†Statistical tests performed: Fisher exact test; Wilcoxon rank-sum test; and χ^2 test of independence.

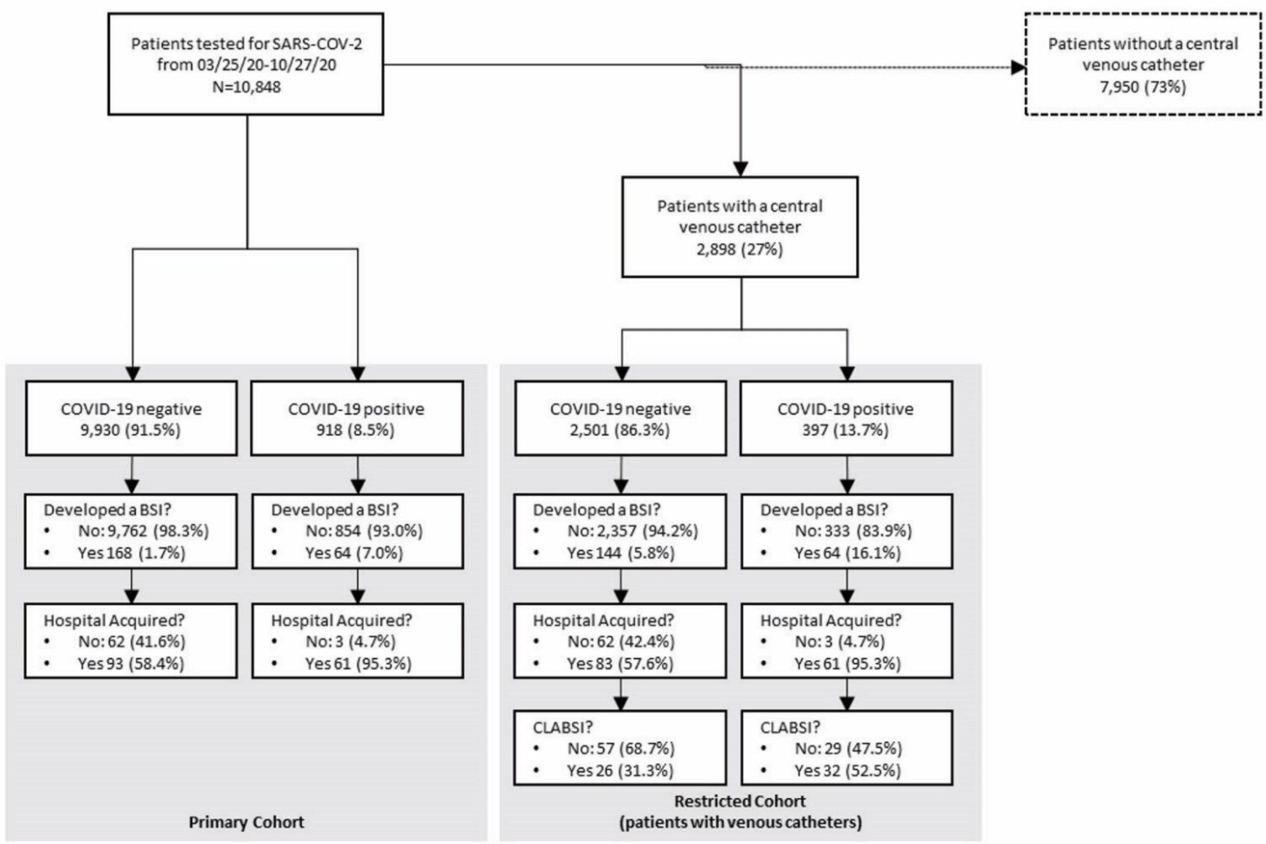

Appendix Figure 1. Flow diagram for selection of patients in study of bloodstream infection risk, incidence, and deaths for hospitalized patients during coronavirus disease pandemic. BSI, bloodstream infection; CLABSI, central line–associated bloodstream infection; COVID-19, coronavirus disease.

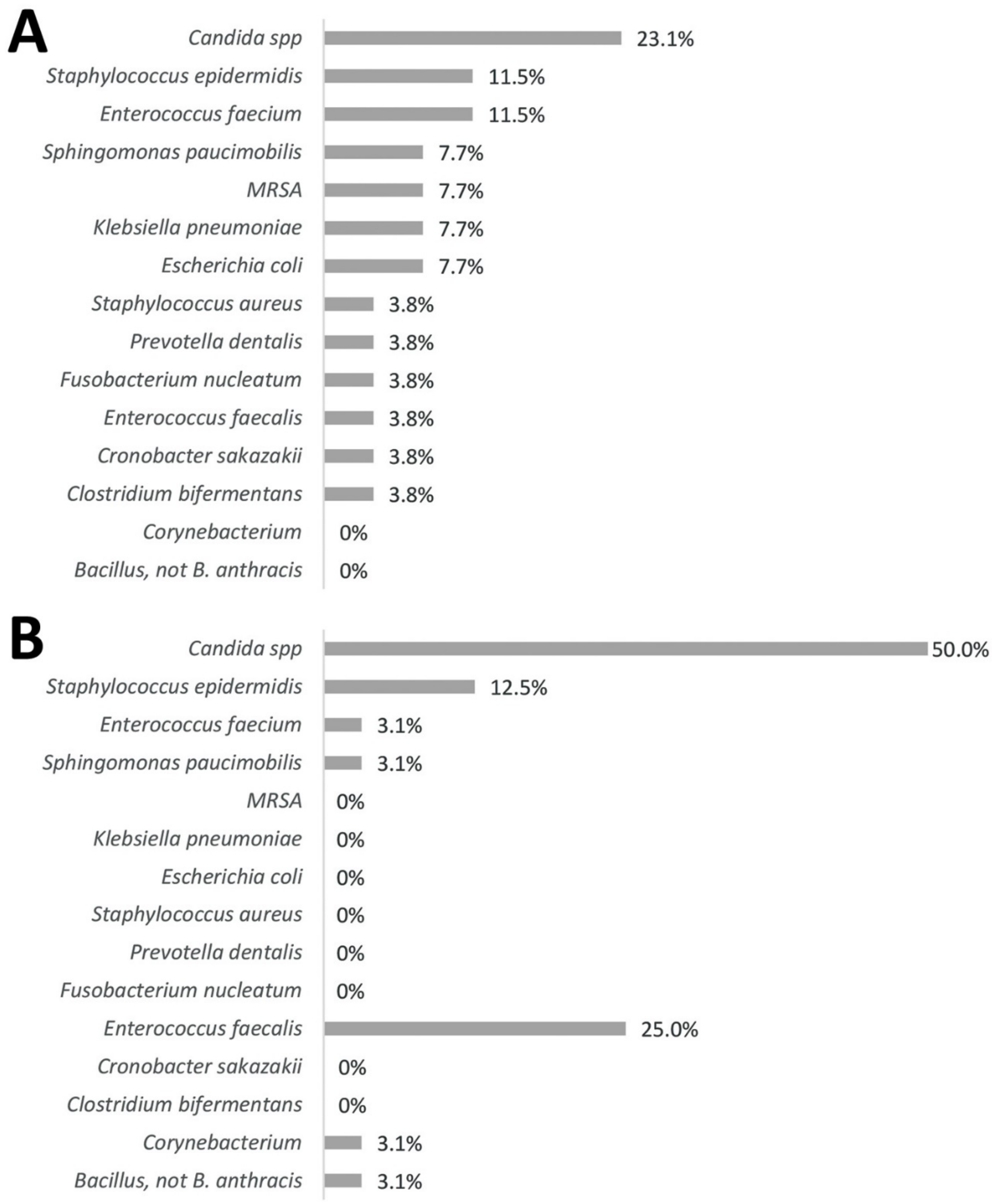

Appendix Figure 2. Organisms responsible for central line–associated bloodstream infections. A) COVID-19–negative patients. B) COVID-19–positive patients. There were 26 COVID-19–negative patients and 32 COVID-19–positive patients who had a central line–associated bloodstream infection. COVID-19, coronavirus disease; MRSA, methicillin-resistant *Staphylococcus aureus.*

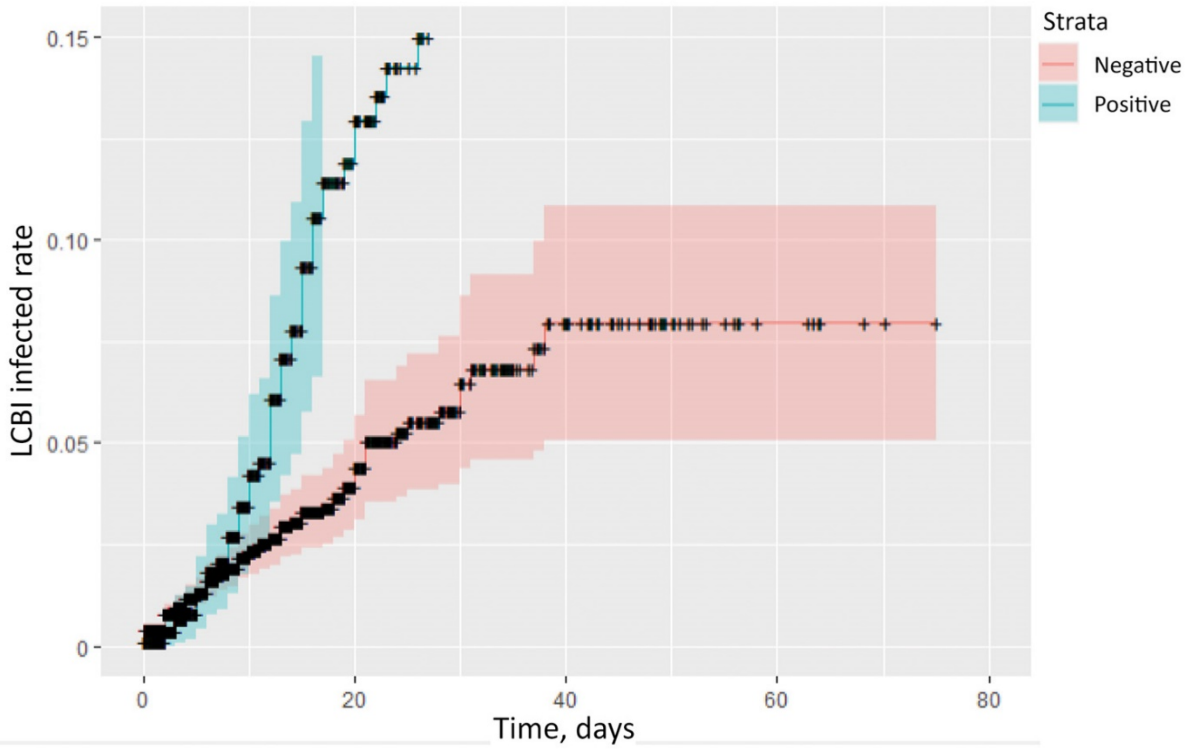

Appendix Figure 3. Cumulative Incidence of LCBI using competing risks models of time-to-infection. Models were adjusted for age, sex, race/ethnicity, payer, body mass index, Elixhauser comorbidity index, sequential organ failure assessment score, and resource use (for details, see Appendix Table 2; time to infection). LCBI, laboratory-confirmed bloodstream infection.

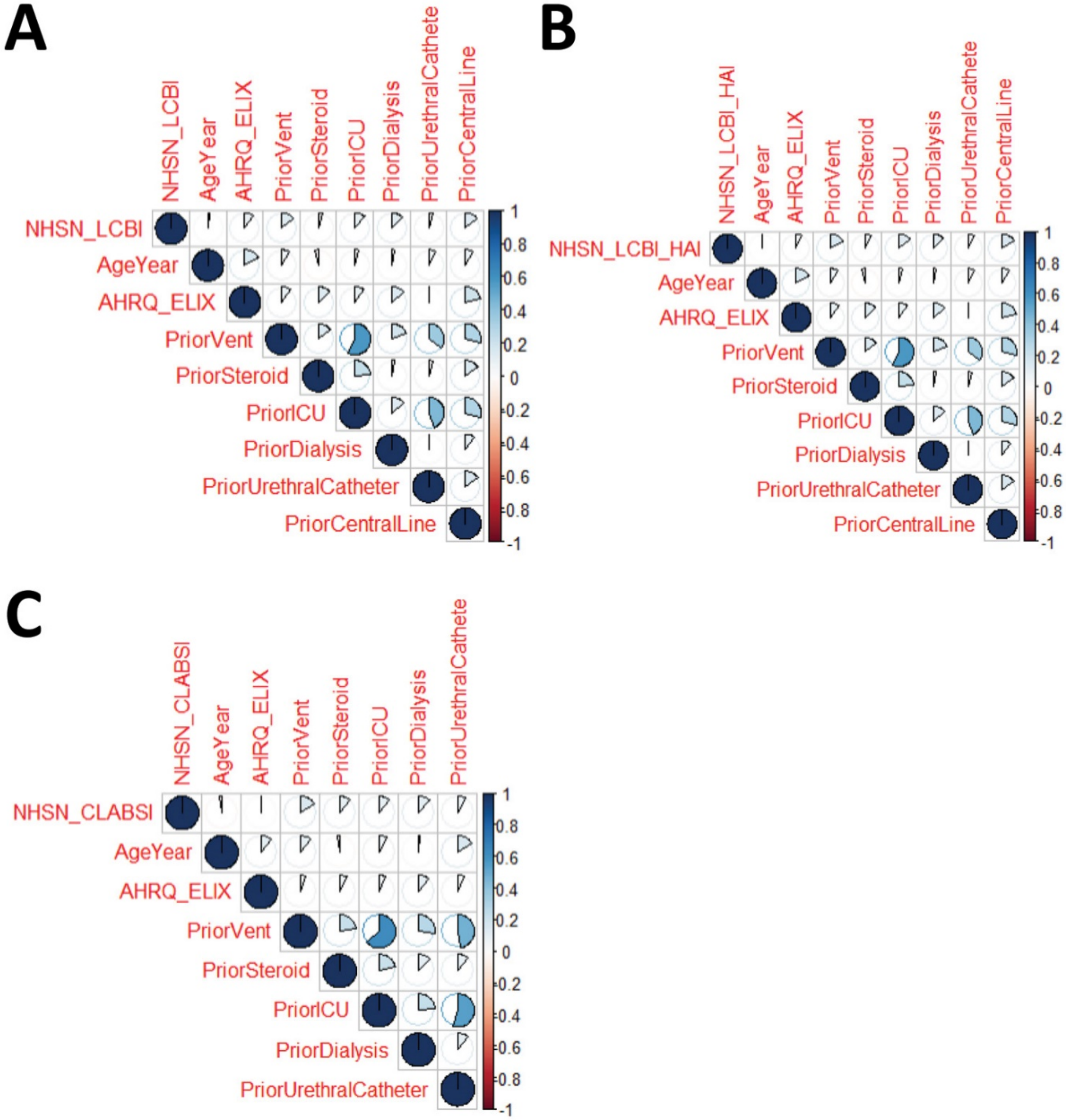

Appendix Figure 4. Evaluation of variables for collinearity using correlation coefficients for LCBI, LCBI HAI, and CLABSI models. AHRQ, Agency for Healthcare Research and Quality; CLABSI, central line–associated bloodstream infection; ELIX, Elixhauser comorbidity measures; HAI, hospital-associated infection; ICU, intensive care unit; LCBI, laboratory-confirmed bloodstream infection; NHSN, National Healthcare Safety Network; vent, ventilation.
